# Vitamin D Status as a Late Pregnancy Biomarker of Perceived Stress

**DOI:** 10.3390/nu17223553

**Published:** 2025-11-14

**Authors:** Maya F. Andrade, Anjali G. Borsum, Mathew J. Gregoski, Myla D. Ebeling, Judith R. Shary, Martin Hewison, Bruce W. Hollis, Carol L. Wagner

**Affiliations:** 1 College of Medicine, Medical University of South Carolina, Charleston, SC 29425, USA; 2 Division of Biostatistics, Department of Public Health Sciences, Medical University of South Carolina, Charleston, SC 29425, USA; gregoski@musc.edu; 3 Division of Neonatology, Department of Pediatrics, Shawn Jenkins Children’s Hospital, Medical University of South Carolina, Charleston, SC 29425, USA; ebelingm@musc.edu (M.D.E.); sharyj@musc.edu (J.R.S.);; 4 Institute of Metabolism and Systems Research, University of Birmingham, Birmingham B15 2TT, UK; m.hewison@bham.ac.uk

**Keywords:** stress in pregnancy, perceived stress, vitamin D, 25-hydroxyvitamin D, maternal health, fetal health, pregnancy, racial disparities

## Abstract

**Background/Objectives**: Stress during pregnancy critically impacts maternal and fetal health. While prior research has linked sociodemographic and biological factors to stress levels, the role of specific biomarkers, such as vitamin D (VD), remains unexplored. This study examined the relationships among sociodemographic factors, VD status (as measured by serum 25-hydroxyvitamin D [25(OH)D] concentration), and perceived stress in pregnant women. We hypothesized that 25(OH)D concentration would be associated with perceived stress levels during pregnancy. **Methods**: A *post hoc* analysis of the Kellogg Pregnancy VD study was conducted on a cohort of 232 pregnant women with Perceived Stress Scale (PSS-10) scores at months 2, 5, and 7 with corresponding 25(OH)D concentrations. PSS-10 scores were classified into two groups: patients with scores of 0–13 were considered to have low stress, while those with scores of 14–40 were considered to have moderate-to-high stress. Logistic regression models identified factors associated with moderate-to-high stress. **Results**: At month 2, univariate analyses showed that being married (*p* = 0.002), having a college education (*p* = 0.0013), and lower BMI (*p* = 0.018) were associated with lower perceived stress, whereas Black race was associated with higher perceived stress (*p* = 0.027). By month 7, higher serum 25(OH)D concentration was the only significant predictor of perceived stress in univariate analysis (*p* = 0.002). In multivariate models at month 7, 25(OH)D approached significance (*p* = 0.053). **Conclusions**: Early in pregnancy, race, marital status, college education, and BMI were significantly associated with PSS-10 score. By month 7, 25(OH)D concentration over time emerged as a factor that was significantly associated in univariate analysis and showed a trend toward significance in multivariate models. VD status, as measured by 25(OH)D concentration, may act as a biomarker of stress during pregnancy. Results warrant further study in prospective intervention trials.

## 1. Introduction

Maternal health during pregnancy plays a vital role in shaping fetal development and influencing long-term health outcomes across the lifespan. Among the numerous factors affecting maternal well-being, psychological stress during pregnancy has emerged as an essential component of both maternal and fetal health outcomes [1,2]. Elevated stress levels have been associated with preterm birth, low birth weight, and impaired neurodevelopmental outcomes [3,4]. These findings underscore the importance of identifying and addressing modifiable factors that contribute to stress during pregnancy.

In parallel, the importance of vitamin D (VD) (cholecalciferol [vitamin D_3_] or ergocalciferol [vitamin D_2_]), a fat-soluble vitamin, in supporting maternal health has gained increasing recognition [5,6,7,8,9,10,11,12,13,14]. While VD is well known for its functions in calcium homeostasis and bone metabolism, growing evidence suggests that it also plays a role in immune function, gene expression, mood regulation, and neuroprotection [15,16,17,18]. 25-hydroxyvitamin D (25(OH)D) is the first metabolite of VD and a biomarker of VD status. Inadequate maternal VD status, as indicated by low serum 25(OH)D concentrations, has been associated with a range of adverse pregnancy outcomes, including preeclampsia, gestational diabetes mellitus, preterm birth, small-for-gestational-age infants, and postpartum depression [5,6,7,8,9,10,11,12,13,14,19,20,21,22,23,24]. The recommended VD supplementation for pregnant women in the United States is 400–600 international units (IU), with most prenatal vitamins typically containing 400 IU of VD, and up to 5000 IU of VD has been considered safe [24,25,26,27,28]. Deficiency of VD (serum 25(OH)D less than 20 ng/mL) and insufficiency (serum 25(OH)D 20–30 ng/mL) is a global public health pandemic, with VD deficiency at 41.4% and insufficiency at 28.9% in the USA from 2001 to 2010 [29], with similar reported results in 2024. Populations at high risk include pregnant women, despite widespread prenatal vitamin use in the US. The dose of 400 IU/day is the same as that recommended for neonates, and recent evidence suggests that a higher daily intake is needed for maternal VD sufficiency [6,27,28]. Furthermore, most pregnant women’s VD status is not measured; yet, in studies where 25(OH)D concentration has been measured, most women are not sufficient, and this has not changed over the last decade. [30,31]. Additionally, prior research has demonstrated an inverse association between 25(OH)D concentrations and psychological stress in nonpregnant populations [32].

Previous work by McLeod et al. has investigated the association between sociodemographic factors, perceived stress, and immune-mediator concentrations [3]. The emphasis was on the relationship between stress and cytokine profiles, suggesting a link between psychosocial stress and immune dysregulation during pregnancy [3]. Although sociodemographic factors and cytokine profiles have been shown to influence psychological stress during pregnancy, the potential role of VD in this context remains largely unexplored [1]. Notably, low 25(OH)D is widespread among pregnant women, particularly those with limited sun exposure, higher body mass index (BMI), and lower socioeconomic status [30,31,33,34,35,36,37].

Given the increased physiological demands for VD during pregnancy, ensuring sufficient levels is essential to promote optimal maternal and fetal outcomes. Despite this, little is known about how biomarkers like 25(OH)D might affect and predict stress levels during pregnancy. While stress and VD status have each been studied independently, the intersection remains poorly researched, representing a significant gap in the prenatal care literature.

This study examines the relationship between sociodemographic factors, VD status, and perceived stress in pregnant women. Rather than relying on treatment group assignment, we assessed VD status using serum 25(OH)D concentrations, recognizing that adherence to supplementation can vary and potentially confound results. Perceived stress was measured using the Perceived Stress Scale (PSS-10), a widely used and globally validated instrument in psychological research [38,39]. We hypothesized that serum 25(OH)D concentration would independently predict perceived stress levels at months 2, 5, and 7 of pregnancy. By evaluating VD status as a potential biomarker for maternal stress, this study aims to generate novel insights that could inform targeted interventions and support the development of comprehensive strategies to improve maternal mental health during pregnancy.

## 2. Materials and Methods

### 2.1. Study Design and Participants

This study was conducted as a *post hoc* analysis of the Kellogg VD Pregnancy Study with the primary goal of preventing health disparities during pregnancy through VD supplementation. The cohort for this secondary analysis was derived from a randomized clinical trial of vitamin D supplementation in pregnancy; the primary trial results have previously been reported [9,10,40]. For the present work, serum 25(OH)D concentration was treated as a continuous predictor of perceived stress, and no analyses of randomized treatment effects were conducted. A total of 405 women participated in a double-blinded and randomized control study to receive either 400 IU or 4400 IU of daily VD supplementation between January 2013 and April 2017 at the Medical University of South Carolina, a regional referral center in the southeastern United States that manages both normal and high-risk pregnancies and performs approximately 3000 deliveries per year. This randomized controlled trial was conducted in accordance with the Declaration of Helsinki and approved by the Institutional Review Board at MUSC (Protocol #20570) and was registered through clinicaltrials.gov (ClinicalTrials.gov, trial #NCT01932788).

### 2.2. Pregnancy Study Design

Participants were enrolled between 10 and 14 weeks of gestation with confirmation of a singleton pregnancy. With written, informed consent, participants took a standard daily prenatal vitamin containing 400 IU of vitamin D_3._ They were randomly assigned to receive either a placebo or 4000 IU, which is the dose found in previous pregnancy studies to achieve VD sufficiency in the form of vitamin D_3_ gummies taken daily (Church & Dwight, Ewing, NJ, USA). The women in this parent study were followed up monthly during pregnancy for nine visits, including the initial recruitment visit, monthly study visits, the delivery visit, and an 18-month postpartum visit for both the mother and child. The primary outcome variable was maternal and neonatal health status as a function of VD status. The study excluded patients with multiple gestations, preexisting calcium disorders, uncontrolled thyroid diseases, uncontrolled parathyroid diseases, and requirements for chronic diuretic or cardiac medication therapy.

### 2.3. Post Hoc Study Design

**Figure 1:**
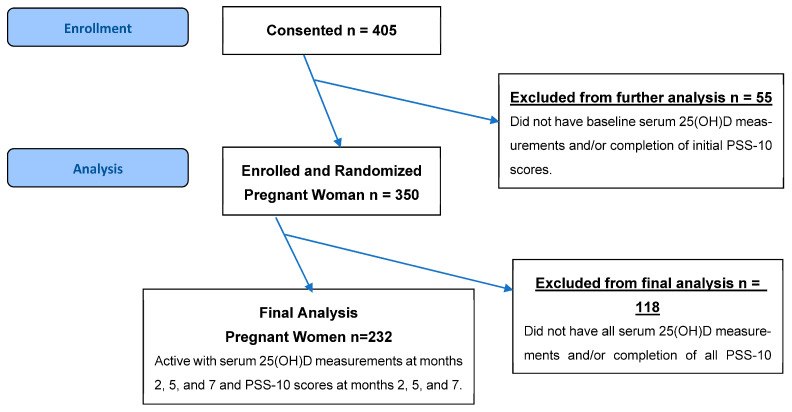
CONSORT flow diagram outlining study procedures.

From the 405 participants in the parent study, our study consisted of 232 patients who had completed the validated 10-question PSS-10 to measure perceived stress at months 2, 5, and 7 of pregnancy, with corresponding 25(OH)D concentrations measured at the same time points. Participants were excluded if they lacked either PPS-10 scores or VD measurements at any of these three study visits, ensuring the final analytic sample remained 232 across all months. The CONSORT flow diagram outlining study procedures is shown in Figure 1. Sociodemographic data, including age, race/ethnicity, marital status, insurance, education, clinical characteristics, and pre-pregnancy BMI, were collected through self-reported questionnaires and verified through medical records when possible. A subgroup of 34 patients with known chronic diseases (including diabetes, hypertension, and BMI > 49) was enrolled.

### 2.4. Perceived Stress Scale Measurement

The PSS-10, a globally validated and widely disseminated questionnaire in psychology, was used to measure stress perception [38,39]. The questionnaire assesses the degree to which situations in one’s life are perceived as stressful, and participants rate each item on a 5-point Likert scale, with total scores ranging from 0 to 40. The scale categorizes scores from 0 to 13 as low stress, 14 to 26 as moderate stress, and 27 to 40 as high stress.

### 2.5. Vitamin D Measurement

Whole blood samples were obtained using EDTA-treated tubes to measure total circulating 25(OH)D concentration. Plasma was separated by centrifugation and stored at −80 °C until analysis. A radioimmunoassay was then performed according to the manufacturer’s protocol (DiaSorin, Stillwater, MN, USA) in the laboratory of Dr. Bruce Hollis.

### 2.6. Statistical Analysis

All statistical analyses were conducted using SPSS version 30 (IBM Corp., Armonk, NY, USA) by a biostatistician who was blinded from previous statistical methods and results related to this trial. Continuous variables were assessed for normality using the Kolmogorov–Smirnov test. Variables with significant departures from normality are reported as medians with interquartile ranges (IQR), while categorical variables are presented as frequencies and percentages. The primary outcome was perceived stress, assessed using the 10-item PSS-10.

Participants were categorized into two groups: low stress (PSS-10 score 0–13) and moderate-to-high stress (PSS-10 score 14–40). Separate logistic regression models were conducted at 2, 5, and 7 months’ gestation to identify predictors of moderate-to-high perceived stress. Candidate predictors included age, race, marital status, education level, BMI, and serum 25(OH)D concentrations. Statistical significance was defined *a priori* at *p* < 0.05.

Variables associated with the outcome at *p* < 0.15 in univariate analyses were entered into multivariate logistic regression models. While no separate power calculation was required as this study reports a secondary observational association rather than a prespecified treatment comparison, an *a priori* power analysis did show an 80% power to detect odds ratios of 1.7 or greater in these models. To assess VD status across pregnancy, we used the area under the curve (AUC) for 25(OH)D, expressed in ng-months/mL, which provides a more comprehensive representation of cumulative VD exposure over time compared to single-time-point measurements.

## 3. Results

A total of 232 participants were included in the study (Figure 1, Table 1). The median maternal age was 29 years with an interquartile range (IQR) of 7 years. The average gestational age was 12 weeks and 4 days with a standard deviation (SD) of 1.6. The cohort was diverse, with 41.8% identifying as White (*n* = 97), 34.5% as Black (*n* = 80), and 22.8% as Hispanic (*n* = 53). The majority of participants were married (71.6%, *n* = 166). Nearly half of the participants were privately insured (47.8%, *n* = 111) and attained a college-level education (46.6%, *n* = 108). The median prepregnancy BMI was 27.1 kg/m^2^ (IQR = 10), and the median baseline serum 25(OH)D concentration was 26 ng/mL (IQR = 13). A subgroup of patients with known chronic diseases, including diabetes (9.1%, *n* = 21), chronic hypertension (3%, *n* = 7), and a BMI greater than 49 (2.6%, *n* = 6), were included. There were 113 patients receiving treatment of 4400 IU VD supplementation and 119 patients receiving a placebo of 400 IU VD supplementation throughout months 2, 5, and 7 (Table 2).

At month 2, among 232 participants, 171 (74%) reported low PSS-10 scores, while 61 (26%) reported moderate-to-high PSS-10 scores. The AUC was 200.7 for low PSS-10 scores and 204.4 for moderate-to-high PSS-10 scores (Table 3). Marital status, educational attainment, and BMI were significantly associated with perceived stress levels. Specifically, participants who were married (*p* = 0.002), had a college education (*p* = 0.0013), and had a lower BMI (*p* = 0.018) were more likely to report lower perceived stress (Table 4). Conversely, Black race was significantly associated with higher perceived stress (*p* = 0.027) (Table 4).

At month 5, 167 participants (72%) reported low PSS-10 scores, and 65 participants (28%) had moderate-to-high PSS-10 scores. The AUC was 204.8 for low PSS-10 scores and 195.0 for moderate-to-high scores (Table 3). Being married (*p* = 0.036) and having a college education (*p* = 0.016) remained significantly associated with lower perceived stress scores (Table 4). Additionally, Black race continued to be associated with higher stress levels (*p* = 0.011) (Table 4).

By month 7, 159 participants (69%) reported low PSS-10 scores, while 73 participants (31%) experienced moderate-to-high PSS-10 scores. The AUC was 212.1 for low PSS-10 scores and 194.9 for moderate-to-high scores (Table 3). At this time point, serum 25(OH)D concentration over time emerged as the only significant independent predictor of perceived stress (*p* = 0.002) (Table 4). Notably, the previously observed association with race was no longer statistically significant.

Multivariate logistic regression analyses were performed at each time point. At month 2, covariates included race, marital status, insurance, education, and BMI; marital status showed a trend toward lower perceived stress (*p* = 0.057). At month 5, with race, marital status, insurance, and education included, having reached the *p* = 0.15 threshold, no covariates were predictive of stress. At month 7, after controlling for marital status, insurance, and education, higher 25(OH)D concentration demonstrated a trend toward significance (*p* = 0.053) (Table 5).

## 4. Discussion

**Figure 2:**
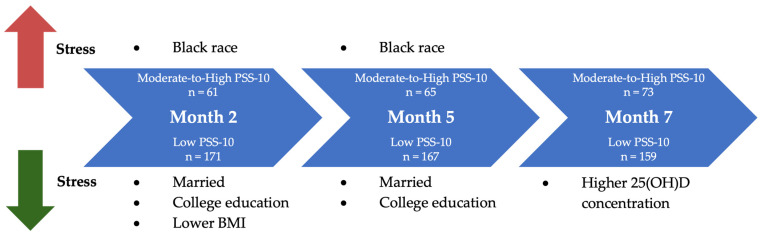
Factors associated with perceived stress in univariate analysis. At month 2, marital status, educational attainment, and lower BMI were significantly associated with lower perceived stress levels, and Black race was significantly associated with higher perceived stress. At month 5, marital status and educational attainment remained significantly associated with lower perceived stress levels while Black race with higher perceived stress. Finally, at month 7, higher serum 25(OH)D concentration over time emerged as the only significant independent predictor of lower perceived stress.

Our study provides novel insights into the dynamic interplay between sociodemographic characteristics, VD status, and perceived stress during pregnancy. Early in pregnancy, race, marital status, college education, and BMI were significantly associated with PSS-10 score. At month 7, however, 25(OH)D concentration over time emerged as a factor that was significantly associated in univariate analysis and showed a trend toward significance in multivariate models (Figure 2).

Based on univariate analyses, in early pregnancy at months 2 and 5, being married and having a college education were associated with lower levels of perceived stress. These findings are consistent with existing literature, highlighting the buffering effects of social support on maternal stress, with married women typically reporting reduced stress levels compared to their unmarried counterparts [41,42,43]. Similarly, higher educational attainment appears to confer protective benefits, potentially through correlates such as improved socioeconomic status, greater access to resources, and enhanced health literacy [44].

There was no association between serum 25(OH)D concentration and perceived stress at months 2 and 5 of pregnancy. This may reflect the complex physiological adaptations of pregnancy, including trimester-specific changes in VD metabolism and stress regulation. Studies have demonstrated that 25(OH)D concentrations may fluctuate during pregnancy, peaking in the late second to early third trimester, influenced by gestational age and season [45,46,47]. Early in pregnancy, these fluctuations may be less pronounced, and the physiological demand for VD increases as pregnancy progresses [45]. The association between serum 25(OH)D concentration and perceived stress may only become apparent in the third trimester due to the combined effects of gestational changes in VD metabolism, increased physiological demands, and evolving stress physiology.

Black race was independently associated with moderate-to-high perceived stress in early pregnancy in univariate analyses. This observation aligns with a substantial body of evidence documenting racial disparities in maternal and neonatal outcomes among Black pregnant women in America [48,49,50,51]. Structural inequities and chronic exposure to stressors likely contribute to this heightened vulnerability. Additional explanations should also be considered. Potential measurement bias, such as cultural differences in the interpretation of the PSS-10, may influence reported stress levels. Residual confounding by unmeasured social or biological factors, as well as heterogeneity within racial/ethnic groups, may also partially account for these findings.

By the third trimester at month 7, sociodemographic predictors were no longer significant. Instead, serum 25(OH)D concentration over time emerged as the sole independent predictor of perceived stress. This supports our initial hypothesis that serum 25(OH)D concentrations would independently predict stress during pregnancy, particularly as physiological demands increase in the last trimester. To our knowledge, this is the first study to demonstrate a relationship between maternal VD status and perceived stress during pregnancy, suggesting a biological mechanism linking VD to stress pathways prominent in pregnancy.

The lack of statistical significance for 25(OH)D in multivariate models likely reflects reduced statistical power rather than a genuine absence of association. Although our overall sample size was 232 participants, stratification across multiple time points and the inclusion of several covariates substantially decreased the number of events per variable in each model. This can attenuate statistical power, widen confidence intervals, and render otherwise meaningful associations nonsignificant. An *a priori* power analysis indicated we had 80% power to detect odds ratios of 1.7 or greater in our logistic models. The consistent directionality and near-significant *p*-values observed in multivariate analyses suggest that 25(OH)D may remain relevant but could not be fully captured given the sample size and analytic framework. Larger studies with greater power are needed to clarify the independent contribution of 25(OH)D to maternal stress physiology.

In addition to the established roles of VD in immune modulation and neuroendocrine regulation, our findings may reflect a broader physiological shift specific to late pregnancy. Previous studies have demonstrated a significant upregulation of the VD metabolic pathway throughout gestation, including a steady rise in VD binding protein from the first through the third trimesters and an early, sustained increase in the active hormone 1,25-dihydroxyvitamin D (1,25(OH)_2_D) until delivery [52,53]. While circulating 25(OH)D does not change markedly during pregnancy except in response to seasonal variation or vitamin D supplementation, prior work has shown a strong correlation between 25(OH)D and 1,25(OH)_2_D, with optimal conversion occurring when 25(OH)D reaches approximately 40 ng/mL [5]. We previously showed the strong correlation between 25(OH)D concentration 1,25(OH)_2_D that exists uniquely in pregnancy and at no other time during the lifecycle [5]. We did not measure 1,25(OH)_2_D concentrations in the present study, and this represents a limitation. Nonetheless, we have previously shown that 25(OH)D is a surrogate for 1,25(OH)_2_D during pregnancy, and data surrounding 1,25(OH)_2_D and its mechanisms of action are germane. This warrants future study to evaluate 1,25(OH)_2_D, particularly given its lower dependence on VD binding protein relative to 25(OH)D, which may serve as a systemic biomarker of reduced stress or enhanced well-being in late pregnancy, with 25(OH)D acting as a surrogate marker.

Previous research in nonpregnant populations has demonstrated an inverse relationship between VD levels and depression, anxiety, and psychological stress [32,48,54]. The extension to pregnancy has been explored in a recent systematic review, which reported that lower maternal 25(OH)D was associated with higher depressive and anxiety symptoms in prenatal and postnatal women [55]. A randomized trial in early pregnancy also reported that daily VD supplementation improved depressive symptoms among deficient women [56]. Prospective cohort data further indicate that antepartum VD deficiency predicts higher postpartum depressive and anxiety symptoms, particularly among women not taking VD supplements, consistent with a threshold-type relationship [57]. These findings align with the association of VD status and perceived stress in the last trimester in our cohort.

At the molecular level, VD acts as a neurosteroid involved in critical brain processes such as neuronal proliferation, apoptosis, and neurotransmission [58,59,60]. VD receptors are widely expressed in brain regions associated with mood and emotion regulation, and deficiency has been linked to depressive symptoms and psychological distress [16,55,56]. Furthermore, studies have proposed that maternal VD levels may influence fetal neurodevelopment and stress reactivity, contributing to long-term mental health trajectories [61]. In addition, VD deficiency may increase activation of the hypothalamic–pituitary–adrenal (HPA) axis, a key mediator of the stress responses, further implicating its role in mood disorders [61]. Emerging pilot data link reduced VD status to altered cortisol patterns in pregnancy, consistent with potential modulation of the HPA axis, and narrative reviews describe VD-dependent regulation of stress-responsive pathways [62,63]. VD also plays an essential role in immune modulation; deficiency can lead to altered cytokine production, increased inflammation, and dysregulated immune responses [64,65,66]. Higher baseline 25(OH)D concentrations in pregnancy are associated with immune-mediatory profiles of healthy pregnancies [10]. The possibility that poor VD status reflects, rather than causes, stress must be considered.

Previous work by McLeod et al. demonstrated that higher perceived stress during pregnancy was associated with increased pro-inflammatory cytokines (IL-2, TNF-α), decreased anti-inflammatory cytokine (IL-10), and lower 25(OH)D concentrations, supporting a link between psychosocial stress and immune dysregulation [3]. Our study extends these findings by identifying VD status over time as a predictor of perceived stress in late pregnancy, suggesting a potential biological mechanism and intervention target distinct from the broader cytokine milieu. Despite its broad physiological significance, VD deficiency and insufficiency remain widespread, especially among pregnant individuals [30].

Concerningly, the role of VD in stress regulation and pathology has been understudied. This is especially important in the context of racial disparities in adverse maternal and birth outcomes among Black pregnant patients. Black women are at higher risk for VD deficiency due to higher melanin levels in the epidermis that reduce VD synthesis following sunlight exposure [67,68]. Furthermore, genetic variations in the VD binding protein gene significantly influence VD levels. Specifically, the “Gc1S” allele, associated with higher circulating VD, is less commonly found in Black patients, which decreases the protein’s binding and transport capacity, leading to lower bioavailability and an increased risk of deficiency [69]. Additionally, racial differences have been observed in the concentrations of immune mediators in pregnant women at baseline and in the second and third trimesters [10]. Given the physiological, psychological, and societal stressors that compound during pregnancy, VD deficiency may exacerbate vulnerability to stress-related complications in this population.

Our findings underscore the potential for VD as a biomarker and modifiable risk factor in maternal stress physiology. This is particularly relevant in light of persistent racial disparities in stress exposure and birth outcomes. Hormonal and metabolic shifts during pregnancy may enhance the sensitivity of stress response systems to VD status, supporting the rationale for targeted interventions. VD supplementation could mitigate these risks and improve both maternal and fetal health outcomes.

The strengths of our study include trimester-specific assessments of both serum 25(OH)D and perceived stress using the globally validated PSS-10, allowing us to track temporal changes across gestation. Another strength is that our cohort also reflects a diverse population of pregnant individuals, with robust baseline and prospective data collection. Further, dietary intake does not confound the study results, as 25(OH)D was used as an objective biomarker that supersedes both the dose of supplementation and diet. As discussed earlier, recent evidence strengthens the biological plausibility of our findings in the framework of VD status and perinatal mood disturbances as well as the VD-HPA axis [55,56,62,63].

Nonetheless, several limitations warrant consideration. First, the *post hoc* nature of the analysis limits the ability to make causal inferences; the results raise questions and should be confirmed in the future. Second, although the PSS-10 is widely validated, it remains a subjective measure influenced by personal interpretation and environmental context. Stress reporting may be misclassified or vary from person to person. Further, unmeasured confounders, including physical activity and pre-existing mental health conditions, could have influenced stress perception. Third, our cohort may not fully represent the general pregnant population, as it was a single-site study with a relatively small sample size, which limits external generalizability. Although our study included White, Black, and Hispanic women, other populations such as Asian, Native American, Pacific Islander, and Middle Eastern women were not represented. Broader inclusion of these groups in future studies will be important to validate our findings and enhance generalizability across diverse populations. Finally, synthesis of intervention trials remains limited; a recent review identified few small randomly controlled trials with mixed findings and emphasized the need for adequately powered studies to test causal effects on perinatal mood and stress [70].

## 5. Conclusions

In conclusion, this study identifies VD status over time, measured as serum 25(OH)D AUC concentration, as a significant and independent predictor of perceived stress in the third trimester of pregnancy in univariate analysis as well as a trend toward significance in multivariate models. While VD may reflect a correlation with stress rather than a causal relationship, our findings provide some evidence for its potential role as a biomarker. Given the exploratory nature of this study, additional validation studies are needed. Previous studies have implicated VD in maternal mental health, which corroborates our findings that VD status remains predictive even after controlling for traditional sociodemographic risk factors. Notably, we observed a shift from social determinants of stress earlier in pregnancy to a biological correlate, 25(OH)D, later in gestation, underscoring the dynamic nature of maternal stress across pregnancy. This pattern suggests that certain interventions may be most effective when timed to specific gestational windows. The third-trimester upregulation of the VD system may reflect an adaptive physiological response that functions as a general anti-stress or well-being signal. Given the high prevalence of VD insufficiency and its disproportionate burden on vulnerable populations, these exploratory findings underscore the importance of future prospective intervention trials to optimize VD status, elucidate underlying biological mechanisms, and assess the potential efficacy of VD supplementation for stress reduction during pregnancy.

## Figures and Tables

**Table 1:** Demographics of Cohort Population.

Sample size (*n*)	232
Maternal Age in Years, median (IQR)	29 (7)
Gestational Age in Weeks and Days, mean (SD)	12w4d (1.6)
Race/Ethnicity: White (%)	97 (41.8%)
Race/Ethnicity: Black (%)	80 (34.5%)
Race/Ethnicity: Hispanic (%)	53 (22.8%)
Marital Status: Married (%)	166 (71.6%)
Insurance: Private (%)	111 (47.8%)
Education: College (%)	108 (46.6%)
BMI, median (IQR)	27.1 (10)
Baseline 25(OH)D in ng/mL, median (IQR)	26 (13)

**Table 2:** Patients receiving 4400 IU versus 400 IU of daily VD supplementation at months 2, 5, and 7.

Month	PSS-10 Score	400 IU	4400 IU
2	Low	82	89
	Moderate-to-High	31	30
5	Low	75	92
	Moderate-to-High	38	27
7	Low	71	88
	Moderate-to-High	42	31

**Table 3:** Maternal VD status represented by AUC, expressed in ng-months/mL, at months 2, 5, and 7.

Month	PSS-10 Score	Median (IQR)
2	Low	200.7 (100)
	Moderate-to-High	204.4 (88)
5	Low	204.8 (101)
	Moderate-to-High	195.0 (85)
7	Low	212.1 (106)
	Moderate-to-High	194.9 (84)

**Table 4:** Univariate Logistic Regression Analyses across Months 2, 5, and 7 ^†^.

Factor	Month 2:			Month 5:			Month 7:		
	OR	*p*	95% CI	OR	*p*	95% CI	OR	*p*	95% CI
Age	0.96	0.220	0.91–1.02	0.95	0.121	0.90–1.01	0.99	0.663	0.93–1.05
Race: Black	2.11	0.027 *	1.09–4.10	2.35	0.011 *	1.22–4.54	1.44	0.266	0.76–2.72
Marital Status: Married	0.37	0.002 *	0.200–0.691	0.521	0.036 *	0.282–0.960	0.61	0.103	0.33–1.02
Insurance: Private	0.57	0.066	0.31–1.04	0.59	0.076	0.33–1.06	0.57	0.051	0.32–1.00
Education: College	0.46	0.013 *	0.25–0.85	0.48	0.016 *	0.27–0.88	0.61	0.091	0.35–1.08
BMI	1.05	0.018 *	1.01–1.08	1.03	0.164	0.99–1.06	1.02	0.283	0.98–1.06
25(OH)D	1.01	0.251	1.00–1.01	0.99	0.133	0.97–1.00	0.97	0.002 *	0.96–0.99
Diabetes	0.87	0.786	0.30–2.47	1.03	0.953	0.38–2.78	1.38	0.494	0.56–3.50
Chronic Hypertension	2.16	0.323	0.47–9.94	0.42	0.425	0.05–3.55	0.87	0.867	0.16–4.58
BMI > 49	2.90	0.200	0.57–14.75	2.65	0.241	0.52–13.46	2.23	0.334	0.44–11.32

^†^ Values reported include odds ratio (OR), * = significant at *p* < 0.05, and 95% confidence interval (95% CI).

**Table 5:** Multivariate Logistic Regression Analyses across Months 2, 5, and 7 ^†^.

Factor	Month 2:			Month 5:			Month 7:		
	OR	*p*	95% CI	OR	*p*	95% CI	OR	*p*	95% CI
Race: Black	1.23	0.588	0.59–2.56	1.79	0.089	0.92–3.50	-	-	-
Marital Status: Married	0.49	0.057	0.23–1.02	0.84	0.639	0.92–3.50	0.79	0.482	0.41–1.53
Insurance: Private	1.26	0.594	0.54–2.94	1.16	0.724	0.51–2.65	0.78	0.552	0.35–1.76
Education: College	0.57	0.193	0.25–1.33	0.52	0.114	0.23–1.17	0.84	0.663	0.38–1.86
BMI	1.03	0.147	0.99–1.07	-	-	-	-	-	-
25(OH)D	1.00	0.655	0.10–1.00	1.00	0.332	1.00–1.00	1.00	0.053	1.00–1.00

^†^ Values reported include odds ratio (OR) and 95% confidence interval (95% CI).

## Data Availability

The original contributions presented in this study are included in the article. Further inquiries can be directed to the corresponding author.

## Abbreviations

The following abbreviations are used in this manuscript:

VD	Vitamin D
PSS-10	Perceived Stress Scale
25(OH)D	25-hydroxyvitamin D
1,25(OH)_2_D	1,25-dihydroxyvitamin D
AUC	Area Under Curve
IQR	Interquartile Range
SD	Standard Deviation
HPA	Hypothalamic–Pituitary–Adrenal
